# High incidence of subsequent re-operation following treatments for medial meniscus tears combined with anterior cruciate ligament reconstruction: second-look arthroscopic study

**DOI:** 10.1186/s43019-019-0009-z

**Published:** 2019-11-01

**Authors:** Sang-Gyun Kim, Soo-Hyun Kim, Jung-Heum Baek, Jae-Gyoon Kim, Ki-Mo Jang, Hong-Chul Lim, Ji-Hoon Bae

**Affiliations:** 1 Department of Orthopaedic Surgery, Korea University Ansan Hospital, 123, Jeokgeum-ro, Danwon-Gu, Ansan‑si, Gyeongki‑do, 15355 Republic of Korea; 2 Department of Orthopaedic Surgery, Korea University Guro Hospital, Korea University College of Medicine, 148, Gurodong-ro, Guro-gu, Seoul, 08308 Republic of Korea; 3 Department of Orthopaedic Surgery, Korea University Anam Hospital, Korea University College of Medicine, 145, Anam-ro, Seongbuk-gu, Seoul, 02841 Republic of Korea; 4 Department of Orthopaedic Surgery, Seoul Barunsesang Hospital, 421, Siheung-daero, Geumcheon-gu, Seoul, 08523 Republic of Korea

**Keywords:** Anterior cruciate ligament, Meniscus, Reconstruction, Repair, Second-look arthroscopy

## Abstract

**Background:**

The Multicenter Orthopaedic Outcomes Network (MOON) group recently reported that medial meniscus (MM) repairs are associated with more frequent re-operations when compared to lateral meniscus (LM) repairs. The purpose of this study was to compare the meniscal healing and the incidence of subsequent re-operation of medial and lateral meniscal tears that occurred concurrently with anterior cruciate ligament (ACL) injuries.

**Methods:**

We retrospectively reviewed patients who underwent second-look arthroscopy after primary ACL reconstruction (ACLR) between June 2005 to December 2016. The healing of meniscal tears following repair or left in situ, and re-tear following partial meniscectomy, were evaluated via second-look arthroscopy and compared between medial and lateral meniscus. Moreover, the incidence of subsequent meniscal re-operation after the index ACLR were investigated and compared between medial and lateral meniscus. Subsequent meniscal re-operation was performed in cases of the following three symptomatic meniscus tears: re-tears at the meniscectomy site; new tears; and failed healing of repaired or left in situ meniscus.

**Results:**

There were 148 meniscal tears in 121 patients at index ACLR. There were 62 MM tears, 38 LM tears, and 24 bilateral meniscus tears. At second-look arthroscopy, the “successful healing” rate for tears following repair was higher in LM tears (91.2%) compared to MM tears (80.0%), although it was not statistically significant (*p* > 0.05). No significant differences were observed in the healing of left in situ tears or re-tear of meniscectomy site between medial and lateral meniscus. Patients with MM tears combined with ACL injuries had a higher incidence of subsequent meniscal re-operation compared to patients with LM tears (25.6% vs 16.1%, *p* = 0.025).

**Conclusions:**

There was a trend for the successful healing rate to be higher in LM repairs than MM repairs. Subsequent meniscal re-operations after ACLR were more frequent in patients with medial meniscal tears concurrently with ACL injuries in comparison to patients with lateral meniscal tears.

**Level of study:**

Level IV, retrospective case series.

## Introduction

Meniscal tears occurring concurrently with anterior cruciate ligament (ACL) injuries are common and can affect treatment outcomes [1, 2]. As the meniscus has an important role for normal knee function, preservation of the meniscus as much as possible during ACL reconstruction (ACLR) has been generally accepted. Success rates of meniscus repair performed concurrently with ACLR can be range in the range of 80–95% [2–4]. Many factors, such as residual laxity, tear length, tear location, patient age, patient physical activity level, and repair integrity, can all affect the biologic healing of meniscal tears and thus clinical outcomes [5, 6]. The management of modifiable risk factors is important to minimize treatment failure.

Recent literature have reported the superior biologic healing potential of the lateral meniscus (LM) compared to the medial meniscus (MM) [1, 7–10]. These studies compared the healing rate of repaired or left in situ meniscus between MM and LM. In addition, they compared the failure of meniscal repairs, defined as repeat surgery on the index meniscus [9, 10]. However, subsequent meniscal re-operation can be required for various reasons. Re-operation can be required because of new meniscal tears (different location or type from index meniscus tear) or re-tear at meniscectomy site. However, there have been limited studies comparing the subsequent meniscal re-operation by these various reasons between the MM and LM.

The purpose was to compare the meniscal healing and the incidence of subsequent re-operation of medial and lateral meniscal tears that occurred concurrently with ACL injuries. Based on the previous study [1], the hypothesis was that healing of the lateral meniscal tears would be superior to the healing of medial meniscal tears and that the incidence of subsequent re-operation would be higher in patients with MM tears.

## Materials and methods

After institutional ethical review board approval, we retrospectively reviewed the patients who underwent second-look arthroscopy after primary ACLR between June 2005 and December 2016. The primary index operation was performed by six surgeons at three branch hospitals of our university medical center. Second-look arthroscopy was performed at least one year after ACLR for reasons including: (1) removal of a tibial fixation screw that caused pain; or (2) treatment of symptomatic meniscal tears. Patients aged > 50 years at the time of the second-look arthroscopy were excluded because of the high possibility of degenerative meniscal tears. Patients with concomitant posterior cruciate ligament injuries were also excluded from the study. The electronic medical records, radiographs, arthroscopic pictures, and videos were reviewed by an orthopedic surgeon who had not been involved in the operation or in the subsequent patient care.

The types of meniscal tear were classified as longitudinal (including bucket handle tears), radial (including oblique and root tears), horizontal, or complex. When one or more types were involved, they were classified as a complex tear. The location of the meniscal tear was represented by three different zones: anterior horn (AH); body (B); and posterior horn (PH). A tear involving more than one zone was defined as an extended tear. The size, location, stability, and reparability of torn meniscus were the determining factors in the type of treatment (repair, meniscectomy, left in situ) that was performed at the time of the index ACLR. Meniscus repair was indicated for acute, unstable longitudinal tears with good tissue quality in the either the red-red or red-white zones and for radial tears that had extended to the red-red zone. We used an outside-in technique for AH tears, an inside-out technique using a double arm needle for body tears, and an all-inside technique using a suture hook for PH tears. Meniscectomy was performed for irreparable tears. Small sized (< 1 cm), partial or full thickness, and stable tears were left in situ.

The healing status of meniscal tears following repair and tears left in situ was evaluated via second-look arthroscopy. Meniscal healing was classified using the modified criteria of Henning et al. [11], which is defined as follows: (1) healed – full-thickness apposition of the original tear with < 10% of the original tear remaining; (2) partially healed – at least 50% of the original tear was healed and was stable when probed; and (3) failed – > 50% of the original tear was present or the presence of unstable meniscus fragments meant additional repairs or resection was required. Healed and partially healed tears were designated as successful healing.

In addition to the rate of meniscal healing, the incidence of subsequent meniscal re-operation after the index ACLR were investigated. Subsequent meniscal re-operation was performed in cases of following three symptomatic meniscus tears: re-tears at the partial meniscectomy site; new tears; and failed healing of repaired or left in situ meniscus.

### Statistical analysis

Demographic variables (including patient sex, age, body mass index [BMI]) and side-to-side differences of condition (including anterior displacement of the tibia, graft, femoral tunnel preparation, meniscal tear characteristics [location, type], and the failure rate of meniscal healing following repair or tear left in situ) were compared between patients who had medial meniscal tears and lateral meniscal tears. Patients who had both medial and lateral meniscal tears at the time of the index ACLR were classified into both the MM group and LM group. An independent t-test was used to compare the continuous variables and a chi-square test was used to compare the categorical variables between the MM and LM groups. Statistical significance was confirmed when the *p* value was < 0.05.

A power analysis was performed to determine the sample size, based on the intergroup difference in subsequent re-operation rate. We used a medium effect size (0.3) of chi-square for goodness-of-fit and contingency. Alpha error was set to 0.05, beta error to 0.80. Finally, we found that more than 122 samples were required for this study.

## Results

There were 148 meniscal tears in 121 patients at the time of the index ACLR (Table 1). The cohort consisted of 62 medial meniscal tears, 38 lateral meniscal tears, and 24 bilateral meniscal tears.

**Table 1:** Demographics of the 148 meniscal tears concurrent to ACL injuries

	Medial meniscus (*n* = 86)	Lateral meniscus (*n* = 62)	*p* value
Age (years)	30 ± 10	27 ± 9	0.086
Sex (Male:Female)	71:15	54:8	0.452
BMI (kg/m^2^)	26 ± 3	25 ± 4	0.308
Follow-up (months)	24 ± 16	22 ± 8	0.414
STSD at second look (mm)	0.9 ± 2.7	1.6 ± 2.2	0.075
Graft (*n*)			0.886
Hamstring autograft	30	19	
BPTB autograft	2	1	
Achilles allograft	9	7	
Tibialis allograft	29	26	
Mixed graft^a^	15	9	
Unknown	1	0	
Femoral tunnel preparation (*n*)			0.944
Transportal	37	27	
Outside-in	25	16	
Transtibial	23	19	
Unknown	1	0	
Meniscus tear type (*n*)			0.003
Longitudinal	67	37	
Radial	4	16	
Horizontal	5	4	
Complex	10	5	
Meniscus tear location (*n*)			0.002
Anterior horn	0	4	
Body	2	11	
Posterior horn	62	39	
A-B	0	0	
B-P	17	7	
A-P	5	1	

Values represent mean ± standard deviation

*BMI* body mass index, *STSD* side-to-side differences, *BPTB* bone patellar tendon bone, *A-B* anterior horn to body, *B-P* body to posterior horn, *A-P* anterior horn to posterior horn

^a^ Hamstring autograft + tibialis allograft

Second-look arthroscopic results after management of meniscal tear are summarized in Table 2. At the time of second-look arthroscopy, the “successful healing” rate for tears following repair was higher in LM tears (91.2%) compared to MM tears (80.0%), although it was not statistically significant (*p* = 0.156). Of 17 meniscal tears left in situ, 64.7% were classified as healed, 17.6% as partially healed, and 17.6% as failed. The “successful healing” rate for tears left in situ was 85.7% for medial meniscal tears and 80.0% for lateral meniscal tears (*p* = 0.761). Overall, the successful healing rate for repaired tears or for tears left in situ was 80.6% for medial meniscal tears and 88.6% for lateral meniscal tears (*p* = 0.261). No significant differences in the demographic variables were seen between patients with successful healing and patients with failed healing (Table 3).

**Table 2:** Second-look arthroscopic results after management of meniscal tear accompanying anterior cruciate ligament reconstruction

	Total	MM tears	LM tears	*p* value
Meniscus repair	94	60	34	0.260
Healed (*n*, %)	78 (83.0)	47 (78.3)	31 (91.2)	
Partially healed (*n*, %)	1 (1.1)	1 (1.7)	0	
Failed (*n*, %)	15 (15.9)	12 (20.0)	3 (8.8)	
Left in situ	17	7	10	0.611
Healed (*n*, %)	11 (64.7)	4 (57.1)	7 (70.0)	
Partially healed (*n*, %)	3 (17.6)	2 (28.6)	1 (10.0)	
Failed (*n*, %)	3 (17.6)	1 (14.3)	2 (20.0)	
Partial meniscectomy	37	19	18	0.630
No re-tear (*n*, %)	34 (91.9)	19 (100.0)	15 (83.3)	
With re-tear (*n*, %)	3 (8.1)	0	3 (16.7)	

*MM* medial meniscus, *LM* lateral meniscus

**Table 3:** Demographic characteristics of patients who had versus did not have successful healing after treatment (repair or conservative) of meniscal tears

	Success healing (*n* = 93)	Failed healing (*n* = 18)	*p* value
Age (years)	29 ± 10	27 ± 11	0.464
Sex (Male:Female)	76:17	14:4	0.695
BMI (kg/m^2^)	25 ± 3	26 ± 5	0.112
Follow-up (months)	24 ± 7	29 ± 28	0.12
STSD at second look (mm)	2.4 ± 2.7	1.8 ± 2.0	0.437
Graft (*n*)			0.165
Hamstring autograft	35	6	
BPTB autograft	0	1	
Achilles allograft	3	2	
Tibialis allograft	39	4	
Mixed graft^a^	15	5	
Unknown	1	0	
Femoral tunnel preparation (*n*)			0.891
Transportal	47	8	
Outside-in	27	6	
Transtibial	19	4	
Meniscal tear compartment (*n*)			
MM tears	35	9	0.598
LM tears	23	4	
Both MM + LM tears	35	5	
Meniscal tear type (*n*)			0.13
Longitudinal	77	13	
Radial	11	2	
Horizontal	2	0	
Complex	3	3	
Meniscal tear location (*n*)			0.283
Anterior horn	3	1	
Body	3	2	
Posterior horn	75	11	
B-P	10	4	
A-P	2	0	
Meniscalus tear treatment (*n*)			
Repair	78	15	0.954
Left in situ	15	3	

Values are presented as mean ± standard deviation unless otherwise specified

*BMI* body mass index, *STSD* side-to-side differences, *BPTB* bone patellar tendon bone, *MM* medial meniscus, *LM* lateral meniscus, *B-P* body to posterior horn, *A-P* anterior horn to posterior horn

^a^ Hamstring autograft + tibialis allograft

On the other hand, patients with MM tears combined with ACL injuries had higher incidences of subsequent meniscal re-operation compared to patients with LM tears (25.6% vs 16.1%, *p* = 0.025). There were 32 subsequent meniscal re-operations for 13 (40.6%) new tears, 16 (50%) in cases of failed healing, and 3 (9.4%) for re-tears following the index ACLR (Table 4). Four repairs and 28 meniscectomies were performed with reference to tissue quality, vascularity, and reparability of the torn meniscus. Four meniscal repairs were performed as follows: two for new tears and two for failed tears. Partial meniscectomies were performed in cases that included: 11 new tears; three re-tears; and 14 failed tears.

**Table 4:** The causes of subsequent re-operation of medial and lateral meniscus

	MM (*n* = 86)	LM (*n* = 62)	*p* value
Subsequent meniscal re-operation (*n*, %)	22 (25.6)	10 (16.1)	0.025
Re-tears	9	7	
Same type and location to previous tears	0	3	
Different type or location to previous tears	9	4	
Failed healing	13	3	

*MM* medial meniscus, *LM* lateral meniscus

## Discussion

The most important finding of this study was that subsequent meniscal re-operations after ACLR were more frequent in patients with medial meniscal tears with ACL injuries in comparison to patients with lateral meniscal tears. The results of this study showed that there was no significant difference in the successful healing rate for repaired tears or for tears left in situ between the MM and LM. However, the incidence of subsequent meniscal re-operations after ACLR were higher in patients with MM tears concurrently with ACL injuries in comparison to patients with LM tears. These results seemed to be due to frequent new tears and failures of meniscal healing in the MM after ACLR compared to the LM.

Recently, the Multicenter Orthopaedic Outcomes Network (MOON) group reported that medial and lateral meniscal tears respond to treatment differently. Current MM repair techniques are associated with more frequent re-operations, worse patient outcomes, loss of joint space, and increased pain when compared to LM repair [1]. The MOON group’s findings that discuss the inferior outcomes after MM injuries have been supported by the recent literature [7, 8]. The Swedish National Knee Ligament Register study found that performance of a MM suture or resection at the time of ACLR was a predictor for non-satisfactory results [7]. The French multicenter study also reported that the risk of recurrence was higher for medial meniscal tears than for lateral meniscal tears at five years after ACLR [8].

Similarly, our study identified a higher rate of failed healing after MM repairs in comparison to the rates of failed healing after LM repairs (20% vs 8.8%), although it was not statistically significant. There are several possible reasons for the difference in healing of the opposing menisci. The MM is inherently less mobile and carries a higher biomechanical load when compared to the LM [12–14]. Since the MM functions as a source of secondary restraint to anterior tibial translation, more stress can be applied to the repaired MM, especially when there is residual laxity after ACLR. This may potentially contribute to more failures of MM repair. Therefore, accurate ACLR, biomechanically stable repair techniques, and biologic stimuli for potentially poor healed meniscal tears, are needed. In addition, the importance of proper rehabilitation after combined meniscus repair and ACLR should be determined in the future.

Clinical symptoms and meniscus re-operation are the most common ways to identify and report meniscus repair failures, as observed from reports in the current literature [3, 9, 10]. However, failed biologic healing after meniscus repair may not represent all of the clinical failures. Several studies have noted that some incomplete or unhealed meniscal lesions at the time of second-look arthroscopy were found to be clinically asymptomatic. Matsushita et al. observed that eight of 19 patients in a meniscal re-tear group following ACLR had no obvious symptoms [15]. Tachibana et al. found that 39.5% of patients who were clinically doing well actually had incomplete or unhealed meniscus repairs at 14.3 months after simultaneous meniscus repair and ACLR [16]. Biologic healing status may not be correlated directly with patient-reported outcomes; therefore, it is questionable whether clinically asymptomatic incomplete or unhealed meniscal lesions should be treated or not during the second-look arthroscopy. In the second-look arthroscopic studies to evaluate meniscal healing, subsequent re-operation rates may have depended upon how many asymptomatic incomplete, or asymptomatic failed lesions, are treated surgically. It seems that surgeons are more likely to treat the unhealed meniscal lesions in order to prevent late symptoms, even though the patient may be not symptomatic at the time of the evaluation. These asymptomatic incomplete or unhealed meniscal lesions cannot be detected during clinical assessment; therefore, further surgical management is generally not planned. However, we should bear in mind that asymptomatic incomplete or unhealed lesions can be potential sources of late symptomatic lesions in the mid to long-term follow-up [17]. Considering that subsequent meniscal re-operation occurred with a significantly higher frequency in patients with concomitant meniscal tears at the time of ACL injury compared to patients with ACL injuries alone, it is important to improve biologic healing of meniscal tears using proper surgical technique, or biologic augmentation, if indicated. In our studies, the rate of re-operation due to failed meniscus repairs was 16%, which is similar to the findings in a recent systematic review reporting clinical failures [3]. All of the failed meniscus repairs except one required re-operation, while none of the partially healed lesions underwent re-operation in our studies. Clinical evaluation alone may underestimate asymptomatic meniscal lesions following ACLR. One may argue that second-look arthroscopic evaluation may lead to unnecessary resection of asymptomatic meniscal tears; however, late identification of meniscal tears decreases the chance of meniscus preservation, which leads to ACL graft failures or degenerative arthritis. Therefore, second-look arthroscopic examination had a clinical relevance for early identification of asymptomatic subsequent meniscal tears.

In our study, 14 patients sustained subsequent new meniscal tears following ACLR. Of those, 11 meniscal tears were incidentally found during second-look arthroscopy concomitant to screw removal. These patients were unable to recall a traumatic event or had no clinical symptoms at all. Meniscectomy was performed in nine patients because the meniscal tears were irreparable and the unstable fragments could potentially have become a source of future symptoms. Similarly, Matsushita et al. [15] observed that eight of 19 patients in their meniscal re-tear group following ACLR had no obvious symptoms. Meniscectomies were performed to prevent future symptoms in all patients. Asahina et al. [17] found that five patients requiring re-operation for failed meniscus repairs had no obvious cause. One possible cause of subsequent meniscal tears in the absence of trauma or sports injury is residual rotational instability despite ACLR [8]. Considering that 8/11 meniscal tears in our study were located at the MM PH, residual rotational instability may have played a role in the repeated shearing forces placed on the MM PH, which is similar to the situation in chronic ACL insufficiency [18–21].

Our study supports that stable meniscal tears can be successfully treated, even if left in situ at the time of ACLR [22–24]. Kyle et al. [22] reported low re-operation rates (3.4%) for meniscal tears untreated at the time of ACLR in an analysis of 208 meniscal tears with a minimum six-year follow-up. The patients’ ages were significantly lower in patients requiring re-operation, while tears measuring > 10 mm more frequently required re-operation. Lee et al. [23] also reported successful healing of stable PH tears of the LM when left in situ at the time of ACLR. In an analysis of 646 meniscal tears by systematic review, 5.4% required re-operation [25]. In addition, they reported a higher rate of re-operation for MM tears that were left in situ (9.5%) compared to the re-operation rates for LM tears left in situ (3.0%). In our study, 3/17 stable meniscal tears (1/7 MM, 2/10 LM) treated by observation only required re-operation. All three tears were longitudinal tears > 10 mm, which supports the report by Kyle et al. [22] Conservative treatment may be a good strategy for small, stable meniscal tears in the peripheral zone, especially in the LM.

This study has several limitations. First, there were limitations to identifying the risk factors for failed meniscal healing, new meniscal tears, re-tears due to a retrospective, non-comparative design, and insufficient information. Although residual rotational instability is generally considered to be a risk factor for inferior biologic healing and subsequent meniscal tears, information on the presence of rotational instability was not available due to the lack of pivot shift grade. Second, selection bias is present because second-look arthroscopy was not performed in all patients who underwent ACLR. Therefore, the re-operation rate for meniscal lesions may be an underestimation of the actual number of failures. Third, tear morphology, including the type, location, and length, was not similar in our studies; therefore, this led to a limitation in comparing the healing results between medial and lateral meniscal tears. Further studies will be required to determine whether similar tears in the MM and LM will exhibit any differences in biologic healing outcomes. Fourth, our study represents the meniscus healing status at an average of two years of follow-up. It is unclear whether completely or partially healed meniscal lesions will remain stable over a longer period.

## Conclusion

There was a trend for the successful healing rate to be higher in LM repairs than MM repairs. Subsequent meniscal re-operations after ACLR were more frequent in patients with medial meniscal tears concurrently with ACL injuries in comparison to patients with lateral meniscal tears.

## Data Availability

The data will not be deposited because they include patients’ personal information.

## Abbreviations

ACL: Anterior cruciate ligament
ACLR: Anterior cruciate ligament reconstruction
AH: Anterior horn
BMI: Body mass index
LM: Lateral meniscus
LMPH: Lateral meniscus posterior horn
MM: Medial and lateral meniscus
MMPH: Medial meniscus posterior horn
MOON: Multicenter Orthopaedic Outcomes Network
MRI: Magnetic resonance imaging
PH: Posterior horn
